# MiR-210 promotes bone formation in ovariectomized rats by regulating osteogenic/adipogenic differentiation of bone marrow mesenchymal stem cells through downregulation of EPHA2

**DOI:** 10.1186/s13018-023-04213-6

**Published:** 2023-10-30

**Authors:** Lijue Ren, Xiaohui Zhu, Jiuting Tan, Xiangyu Lv, Jiahui Wang, Fei Hua

**Affiliations:** 1 Department of Endocrinology, The Third Affiliated Hospital of Soochow University, Changzhou, 213100 Jiangsu China; 2 Department of Endocrinology, The First Affiliated Hospital of Baotou Medical College, Inner Mongolia University of Science and Technology, Baotou, 014010 Inner Mongolia China

**Keywords:** miR-210, Bone marrow mesenchymal stem cells, Osteogenic/adipogenic, Ovariectomized rats

## Abstract

**Purpose:**

In osteoporosis, the balance between osteogenic and adipogenic differentiation of mesenchymal stem cells (MSCs) is disrupted. The osteogenic differentiation of bone marrow MSCs (BMSCs) is important for improving osteoporosis. The aim of this study was to explore the role and molecular mechanism of miR-210 in the balance of osteogenic/adipogenic differentiation of BMSCs in postmenopausal osteoporosis.

**Methods:**

Postmenopausal osteoporosis rat models were constructed by ovariectomy (OVX). BMSCs were isolated from the femur in rats of Sham and OVX groups. MiR-210 was overexpressed and suppressed by miR-210 mimics and inhibitor, respectively. Quantitative real-time polymerase chain reaction (qRT-PCR) was used to detect the relative mRNA expression of miR-210, ephrin type-A receptor 2 (EPHA2), alkaline phosphatase (ALP), osterix (OSX), osteocalcin (Bglap), Runt-related transcription factor 2 (Runx2), peroxisome proliferator activated receptor gamma, and fatty acid binding protein 4 (FABP4) in each group of rat femoral tissues or BMSCs. Western blot was applied to detect the protein expression level of EPHA2 in rat femoral tissues and cells. Alizarin red S staining and oil red O staining were performed to assess the osteogenic and adipogenic differentiation of BMSCs, respectively. In addition, the targeting relationship between miR-210 and EPHA2 was verified by a dual luciferase gene reporter assay.

**Results:**

The expression of miR-210 was significantly reduced in femoral tissues and BMSCs of OVX rats, and its low expression was associated with reduced bone formation. The osteogenic differentiation was enhanced in OVX rats treated with miR-210 mimic. Overexpression of miR-210 in transfected BMSCs was also found to significantly promote osteogenic differentiation and even inhibit adipogenic differentiation in BMSCs, while knockdown of miR-210 did the opposite. Further mechanistic studies showed that miR-210 could target and inhibit the expression of EPHA2 in BMSCs, thus promoting osteogenic differentiation and inhibiting adipogenic differentiation of BMSCs.

**Conclusion:**

MiR-210 promotes osteogenic differentiation and inhibits adipogenic differentiation of BMSCs by down-regulating EPHA2 expression. As it plays an important role in the osteogenic/adipogenic differentiation of osteoporosis, miR-210 can serve as a potential miRNA biomarker for osteoporosis.

**Supplementary Information:**

The online version contains supplementary material available at 10.1186/s13018-023-04213-6.

## Introduction

Osteoporosis (OP) is a systemic disease characterized by reduced bone mass and destruction of bone microstructure, which puts patients at a high risk of fragility fractures [1]. OP consists of two main categories: primary and secondary, and the most common primary OP is postmenopausal OP (PMOP) [2]. PMOP is a consequence of a significant decrease in estrogen levels after menopause and a subsequent serious imbalance between osteoblast-mediated bone formation and osteoclast-mediated bone resorption. The imbalance leads to a decline in bone mass, changes in bone microstructure, and even fragility fractures [3]. The disorder of bone metabolism and fragility fractures caused by PMOP reduce the quality of life of millions of people, limit patient autonomy, increase disability and shorten life expectancy, while also imposes a huge economic burden [4]. Presently, the main methods for treating OP involve preventing bone resorption and encouraging bone growth. Anti-OP drugs Bisphosphonates, Denosumab, Romosozumab, Ibandronate, pamidronate, zoledronate and Raloxifene could increase bone BMD and reduce fracture rate in postmenopausal women with OP to varying degrees [5–8]. However, these agents produce limited therapeutic efficacy and even have side effects [6]. Not only that, there are few drug options available. Hence, the search for novel OP treatment strategies is crucial.

Increasing the formation of new bone while preventing the absorption of old bone is one way to prevent OP. Bone marrow mesenchymal stem cells (BMSCs) are spindle-shaped adherent cells capable of forming single cell-derived clones. Widely present in the bone marrow cavity of the cancellous bone of axial skeleton and peripheral bones, BMSCs are ideal seed cells for tissue engineering because of their characteristics of self-renewal and differentiation into multiple cell types [9]. Osteogenic differentiation of BMSCs is vital for improving OP, and the differentiated cells are important effectors in regulating the cellular osteogenic process and critical contributors to the production of extracellular matrix and mineralization [10]. BMSCs have been extensively applied in bone diseases in both animal models and humans, including osteoarthritis, fractures, and OP. Studies have determined that the proliferation and osteogenic differentiation of mesenchymal stem cells in OP patients are reduced, but this condition is reversed by transplantation of BMSCs [11–13]. Undoubtedly, BMSCs have created a novel approach to treating OP. However, osteogenic/adipogenic differentiation of BMSCs is a complex process influenced by many factors and thus regulating this process is crucial for the clinical application of BMSCs.

MicroRNAs (miRNAs), a class of endogenous non-coding RNAs of 18–25 nucleotides in length, regulate gene expression by specifically binding to the 3′-UTR of target genes. They are considered to be an important regulatory molecule in epigenetics, and involved in the biological activities of many diseases, such as cell differentiation, proliferation, invasion, metastasis, and apoptosis [14]. Also, a great deal of research has shown that miRNAs are crucial regulators of bone metabolism and are essential in preserving the homeostasis of bone metabolism, particularly when it comes to controlling the differentiation of BMSCs into particular lineages [15, 16]. Oliviero et al. also showed that there are complex interactions between miRNAs and their multiple target genes, and these interactions may play an important role in gene regulation of OA and control of homeostasis pathways [17]. Li et al. showed that miR-291a-3p activated the Wnt/β-catenin signaling pathway by directly inhibiting DKK1 expression to promote osteogenic differentiation of BMSCs [18]. MiRNA-210 (miR-210) is one of the miRNAs and has been reported to maintain the properties of BMSCs [19, 20]. In addition, a sequencing study by Gu et al. based on clinical samples from OP patients showed that miR-210-3p was down-regulated in the femur of OP patients and may be involved in bone development, bone marrow cell proliferation, osteoblast development, negative regulation of osteoblast differentiation and osteoclast development, as well as several osteogenesis-related pathways [21]. Our previous published studies suggested that high expression of miR-210 improved bone tissue micromorphology, regulated bone formation and absorption to alleviate postmenopausal osteoporosis, possibly by activating the VEGF/Notch1 signaling pathway [22]. However, the exact mechanism by which it works is unclear. Based on this, we aimed to investigate the role of miR-210 in the osteogenic/adipogenic differentiation balance of BMSCs in postmenopausal OP and its molecular mechanism. Firstly, we constructed menopausal OP rat models, and isolated BMSCs from rat femurs for cell culture. MiR-210 in the cells was then overexpressed by transfecting with lentivirus. Quantitative real-time polymerase chain reaction (qRT-PCR), western blot, alizarin red S staining, oil red O staining, and dual luciferase reporter gene assay were performed during the process of achieving the aim.

## Materials and methods

### Establishment and grouping of animal models

All animal experiments were approved by the Ethics Committee of The Third Affiliated Hospital of Soochow University (C202212-2) and conducted in accordance with the approval guidelines. All procedures were performed under pentobarbital sodium anesthesia and every effort was made to minimize pain. Twenty-six healthy six-week-old female Sprague Dawley (SD) rats were purchased from Shanghai Model Organisms Center, Inc. The rats were housed for 1 week under acclimatization at a relative humidity of approximately 50–60%, a temperature of 22 °C, and a 12 h:12 h light/dark cycle, during which they had a free ingestion of food and water. OVX-induced OP rat models were established by referring to the method in the study by Khedgikar et al. [23]. Specifically, the rats were anesthetized by intraperitoneal injection of 50 mg/kg pentobarbital sodium; their bilateral ovaries were exposed through midline incisions in the dorsal skin and muscle layers, and then were removed after ligation of the cornua uteri. Finally, the muscle incisions were sutured, and the skin was closed.

The negative mimic and miR-210 mimic were designed and synthesized by Guangzhou RiboBio Co., Ltd. The 26 rats were randomly divided into two groups: Sham group (*n* = 8): surgical procedures in this group were consistent with those in the OVX group, except that the ovaries were not removed; OVX group (*n* = 18): OVX surgery was performed in this group. Subsequently, the rats in the OVX group were further divided at random into three groups: the OVX group, the OVX + NC mimic group, and the OVX + miR-210 mimic group. After four weeks of postoperative recovery, 5 μL of saline was injected into the tail vein of rats in the Sham and OVX groups once a week for 8 weeks; 5 μL of negative control mimic (NC mimic) and miR-210 overexpression mimic (miR-210 mimic) were injected into the tail vein of rats in the OVX + NC mimic and OVX + miR-210 mimic groups once a week for 8 weeks, respectively. At the end of treatment, all rats were killed through cervical dislocation and the femur was isolated under aseptic conditions for subsequent experiments.

### Isolation and identification of bone marrow mesenchymal stem cells

Three rats were randomly selected from each of the Sham and OVX groups, and BMSCs were isolated from rat femurs referring to the previous study [24]. The extracted cells from the two groups were also named as Control group and OVX group, respectively. In brief, 5 ml of DMEM medium (Hyclone, USA) containing 10% FBS (Gibco, USA) and 500 μl of heparin (HY-17567 MedChemExpress, USA) was used to wash the marrow cavity of the femur several times. The obtained washing solution was mixed well and then placed in DMEM medium containing 100 × cytochalasin (Solarbio, Beijing, China) and 10% FBS for adherent culture. When the cell density reached about 90%, the cells were passaged.

The surface markers (CD29, CD90, CD11b) of third passage rat BMSCs were detected by flow cytometry. The BMSCs were collected and resuspended in phosphate-buffered saline (PBS) to achieve 1 × 10^6^ cell density. Then CD29-fluorescein Isothiocyanate (FITC) (11-0291-82), CD90-allophycocyanin (APC) (17-0900-82) and CD11b-phycoerythrin (PE) (12-0110-80) monoclonal antibodies (eBioscience, Inc., San Diego, CA, USA) were added to the suspension and incubated at room temperature for 30 min for flow cytometry.

### Transfection grouping of bone marrow mesenchymal stem cells

The negative inhibitor and miR-210 inhibitor, vector and pcNDA3.1-EPHA2 were designed and synthesized by Guangzhou RiboBio Co., Ltd. The 2–5 generations of BMSCs isolated from the Control group were subjected to transfection and then grouping. Initially, the BMSCs were inoculated in 6-well plates and treated differently in groups when the cell density developed to 70% according to the following protocol. NC inhi group: BMSCs were transfected with negative inhibitor; miR-210 inhi group: BMSCs were transfected with miR-210 inhibitor; NC mimic group: BMSCs were transfected with negative mimic; miR-210 mimic group: BMSCs were transfected with miR-210 mimic; NC mimic + vector group: BMSCs were transfected with negative mimic and negative overexpression vector; miR-210 mimic + vector group: BMSCs were transfected with miR-210 mimic and negative overexpression vector; NC mimic + EPHA2 group: BMSCs were transfected with negative mimic and EPHA2 overexpression vector (pcNDA3.1-EPHA2); miR-210 mimic + EPHA2 group: BMSCs were transfected with miR-210 mimic and pcNDA3.1-EPHA2. Lipofectamine 2000 transfection reagent (Invitrogen, USA) was adopted for each transfection. The transfected BMSCs were collected 48 h after transfection for further analysis.

### Osteogenic and adipogenic differentiation of bone marrow mesenchymal stem cells

The transfected BMSCs were inoculated in culture dishes. Subsequently, the cells were cultured in osteogenic differentiation medium (DMEM medium supplemented with 10 mM of β-glycerophosphate, 10 nM of dexamethasone, 50 μg/ml of ascorbic acid, 10% FBS and 100 × penicillin) and adipogenic differentiation medium (DMEM medium containing 10 μM of insulin, 100 nM of dexamethasone, 500 μM of 3-isobutyl-1-methylxanthine, 200 μM of indomycin, 10% FBS and 100 × penicillin) for 10–14 days to induce osteogenic differentiation and adipogenic differentiation, respectively [25, 26].

### Quantitative real-time polymerase chain reaction (qRT-PCR)

Total RNA from femoral tissues or BMSCs was extracted using Trizol reagent (Solarbio, Beijing, China), and the concentration of the extracted RNA was determined using Nanodrop software. Then cDNA was synthesized by reverse transcription of RNA using the PrimeScript RT kit (Takara, Japan). Next, the expression of miR-210, ephrin type-A receptor 2 (EPHA2), alkaline phosphatase (ALP), osterix (OSX), osteocalcin (Bglap), Runt-related transcription factor 2 (Runx2), peroxisome proliferator activated receptor gamma (PPARG), and fatty acid binding protein 4 (FABP4), as well as internal controls U6 and GAPDH in cDNA was detected using TB Green-based qPCR kit (Takara, Japan). The primer sequences synthesized by Tsingke Biotechnology Co., Ltd. are shown in Table 1. The qPCR data of the above coding genes and miR-210 were normalized with the expression of GAPDH and U6 as internal controls, and the resulting data were analyzed by the 2^−ΔΔCt^ method [27].

**Table 1:** qRT-PCR primer sequences

Genes	Primer sequences
ALP	F: 5′-CCTGGACCTCATCAGCATTT-3′
	R: 5′-AGGGAAGGGTCAGTCAGGTT-3′
OSX	F: 5′-CACTCTCCCTGCCAGACCTC-3′
	R: 5′-GCCATAGTGAACTTCCTCCTCAAG-3′
Bglap	F: 5′-TGCTCACTCTGCTGACCCTG-3′
	R: 5′-TTATTGCCCTCCTGCTTG-3′
Runx2	F: 5′-CTACTCTGCCGAGCTACGAAAT-3′
	R: 5′-TCTGTCTGTGCCTTCTTGGTTC-3′
PPARG	F: 5′-TGGAGCCTA AGTTTGAGTT-3′
	R: 5′-CAATCTGCCTGAGGTCTG-3′
Fabp4	F: 5′-CCCAGATGACAGGAAAGTGAA-3′
	R: 5′- TCACGCCTTTCATGACACA-3′
miR-210	F: GGAGATCTGACCAGGTCATTTGCATAC
	R: GGGAATTCGATATGACCACACC TGTG
EPHA2	F: 5′-GCCAGCGATĞTGTGGAGCTA-3′
	R: 5′-AGCCGTCGTTGATGGCTTTC-3′
U6	F: 5′-GCTTCGGCAGCACATATACTAAAAT-3′
	R: 5′-CGCTTCACGAATTTGCGTGTCAT-3′
GAPDH	F: 5′-AACGACCCCTTCATTGACCTC-3′
	R: 5′-CCTTGACTGTGCCGTTGAACT-3′

### Alizarin red S staining and quantitative analysis

BMSCs in each group were cultured in the osteogenic differentiation medium for 12–14 days and then stained with alizarin red. Specifically, the osteogenic differentiated BMSCs were washed twice with PBS after removing the medium, and then fixed by adding fixative for 20 min. After discarding the fixative and washing the cells three times with PBS, appropriate amount of Alizarin Red S staining solution was added to cover the cells uniformly at room temperature for 30 min. Later, the cells were washed using ddH_2_O, observed and photographed. Finally, the quantitative analysis was carried out by eluting the alizarin red dye with 10% cetyl pyridine chloride (Solarbio, Beijing, China), and the optical density value (OD) at 570 nm of each group was measured.

### Oil red O staining and quantitative analysis

BMSCs in each group were cultured in adipogenic differentiation medium for 12–14 days and then received oil red O staining. Speaking, the medium was removed and the differentiated BMSCs were washed with PBS. Upon the addition of 4% paraformaldehyde (P0099, Beyotime, Shanghai, China) for 10 min, the cells were washed twice with PBS. The obtained cells were first covered with an appropriate amount of staining scrubbing solution for 20 s and then removed, followed by staining with an appropriate amount of oil red O staining solution for 20 min. The oil red O staining solution was removed, the appropriate amount of staining washing solution was added, and they were left to stand for 30 s. Following that, the staining scrubbing solution was removed, and the cells were washed with PBS for 20 s. The nuclei were re-stained with hematoxylin staining solution (C0107, Beyotime, Shanghai, China) for 1–2 min. Again, PBS was employed to wash the cells three times. With appropriate amount of PBS adding to cover the cells, they were observed under a microscope (Olympus) and then photographed. As for quantitative analysis, the OD at 490 nm of each group was measured through adding 100% isopropanol to elute oil red O.

### Dual luciferase reporter assay

Before transfection, 293 T cells were inoculated in 6-well plates and cultured until the fusion level reached 80%. The cells were cotransfected with the constructed EPHA2 wild type (EPHA2-WT) or mutant type (EPHA2-MUT) vector and miR-210 mimic or NC mimic. Moreover, the cells were continued to be cultured for 48 h after the transfection. Later, the resulting cells were lysed at room temperature for 20 min, followed by centrifugation. The supernatant was extracted, kept at -20 ℃, and added to the luciferase substrate. The luciferase activity was detected by chemiluminescence apparatus, and the relative firefly luciferase activity was calculated using the Ranilla luciferase activity as internal control.

### Western blot

Femoral tissue or BMSCs were lysed for 20 min using RIPA lysis solution (P0013B, Beyotime, China) supplemented with 1 mM of the protease inhibitor phenylmethylsulfonyl fluoride (PMSF, ST506, Beyotime, China). The cells were later broken up by ultrasonication in an ice bath, fully lysed, and centrifuged at 12,000*g* for 10 min. The concentration of total protein in supernatant was determined using the BCA protein assay kit (P0012S, Beyotime, China). After that, the total protein was added with 5 × SDS-PAGE protein loading buffer and together boiled. With the help of SDS-PAGE gel electrophoresis, 30 μg of total protein was separated and transferred onto 0.45 μm of polyvinylidene fluoride membranes (PVDF, Millipore) via electro transfer. The membranes were blocked with a blocking buffer containing 5% skimmed milk powder for 1–3 h. After washing three times with PBST, they were incubated with anti-EPHA2 (37-4400, Invitrogen. USA) or anti-β-actin (ab6276, Abcam, UK) overnight at 4 °C on a shaker. Later, the membranes were incubated with diluted secondary antibody (ZB-2305, ZSGB-BIO, China) for 1 h at room temperature following a rinse with PBST solution for 10 min × 3 times. The PBST rinse was performed again, and ultrasensitive ECL chemiluminescent solution (P0018FS, Beyotime, China) was added uniformly and dropwise. The membranes were then exposed to the FliorchemHD2 imaging system and the bands were collected. The grayscale value of each band was analyzed by ImageJ software, and the relative expression level of EPHA2 was calculated using the grayscale value of β-actin as an internal control [28].

### Statistical analysis

All data were expressed as mean ± standard deviation (SD). Graphpad Prism 9.0 software was used to visualize and statistically analyze the results. T-test was applied for comparison between two groups, and one-way ANOVA for comparison between multiple groups. *P* < 0.05 was used as the criterion for determining significant differences.

## Results

### MiR-210 expression is down-regulated in femoral tissues of ovariectomized rats and promotes osteogenesis

**Fig. 1:**
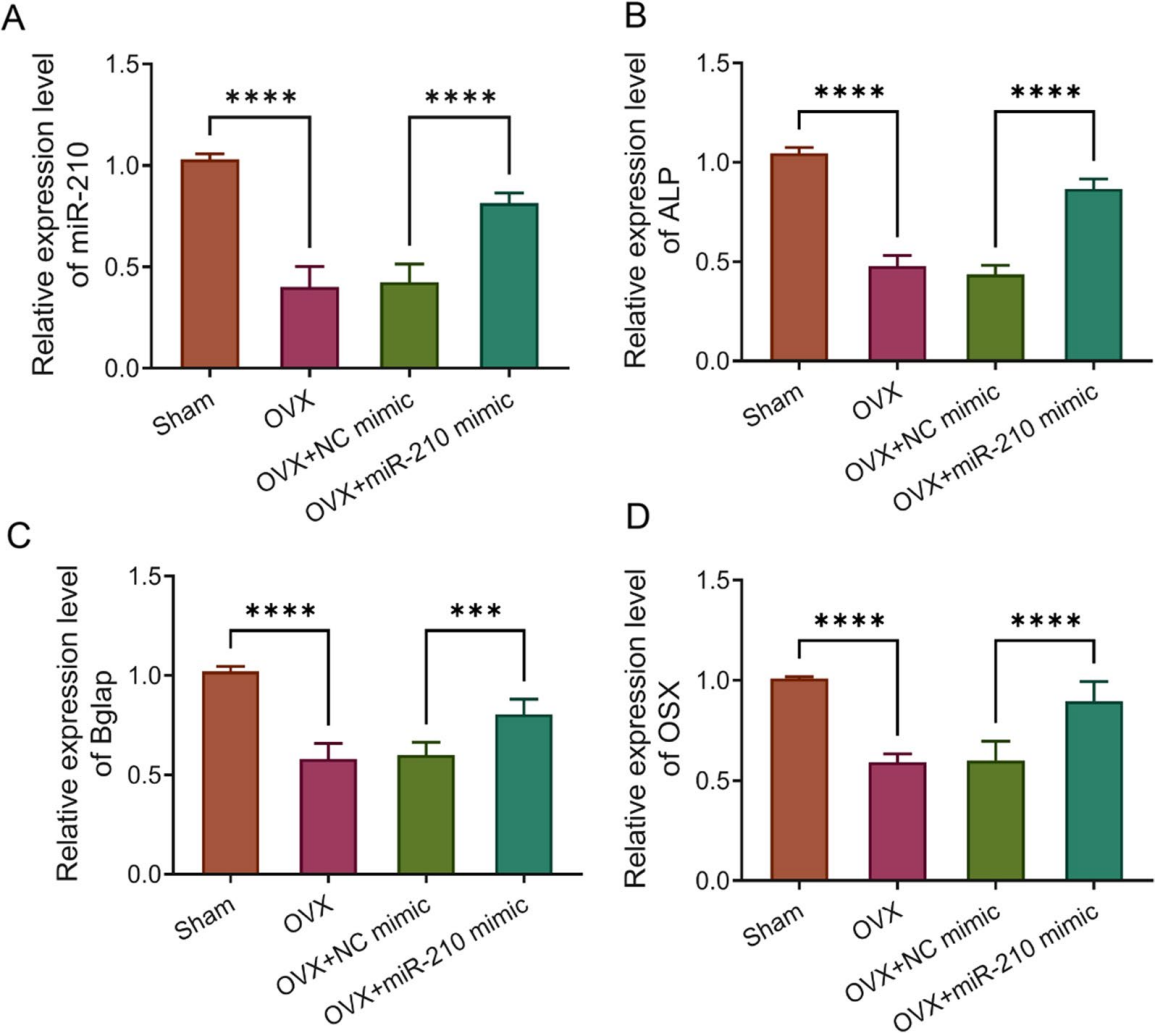
MiR-210 expression is down-regulated in femoral tissues of OVX rats and promotes osteogenesis. **A**–**D**, qRT-PCR was performed to detect the relative mRNA expression levels of miR-210 (**A**) and bone-formation related genes ALP (**B**), Bglap (**C**) and OSX (**D**) in femoral tissues of each group, *n* = 5. ***P < 0.001, ****P < 0.0001. OVX: ovariectomy; qRT-PCR: quantitative real-time polymerase chain reaction; ALP: alkaline phosphatase; Bglap: osteocalcin; OSX: osterix

To explore the role of miR-210 in postmenopausal OP rats, we constructed postmenopausal OP rat models by OVX, which received 8 weeks of continuous miR-210 mimic tail vein injection to overexpress miR-210. The results of qRT-PCR showed that the expression level of miR-210 in femoral tissues of the OVX group was significantly lower than that of the Sham group, while the expression of miR-210 in the femur of rats in the OVX + miR-210 mimic group was significantly higher than that in the OVX + NC mimic group (Fig. 1A). We then examined the expression of genes related to osteogenic markers in the femoral tissues of rats in each group. It was discovered that the gene expression levels of osteogenic markers ALP, Bglap, and OSX were much lower in the femoral tissues of rats in the OVX group than those in the Sham group (*P* < 0.0001). In addition, we also found that miR-210 mimic treatment significantly increased the gene expression levels of ALP, OSX and Bglap in femoral tissues of OVX rats, but there was no significant difference in these levels between the OVX group and the OVX + NC group (Fig. 1B–D). The above results confirmed the promotion role of miR-210 in osteogenesis in OVX rats.

### MiR-210 promotes osteogenic differentiation and inhibits adipogenic differentiation in bone marrow mesenchymal stem cells

**Fig. 2:**
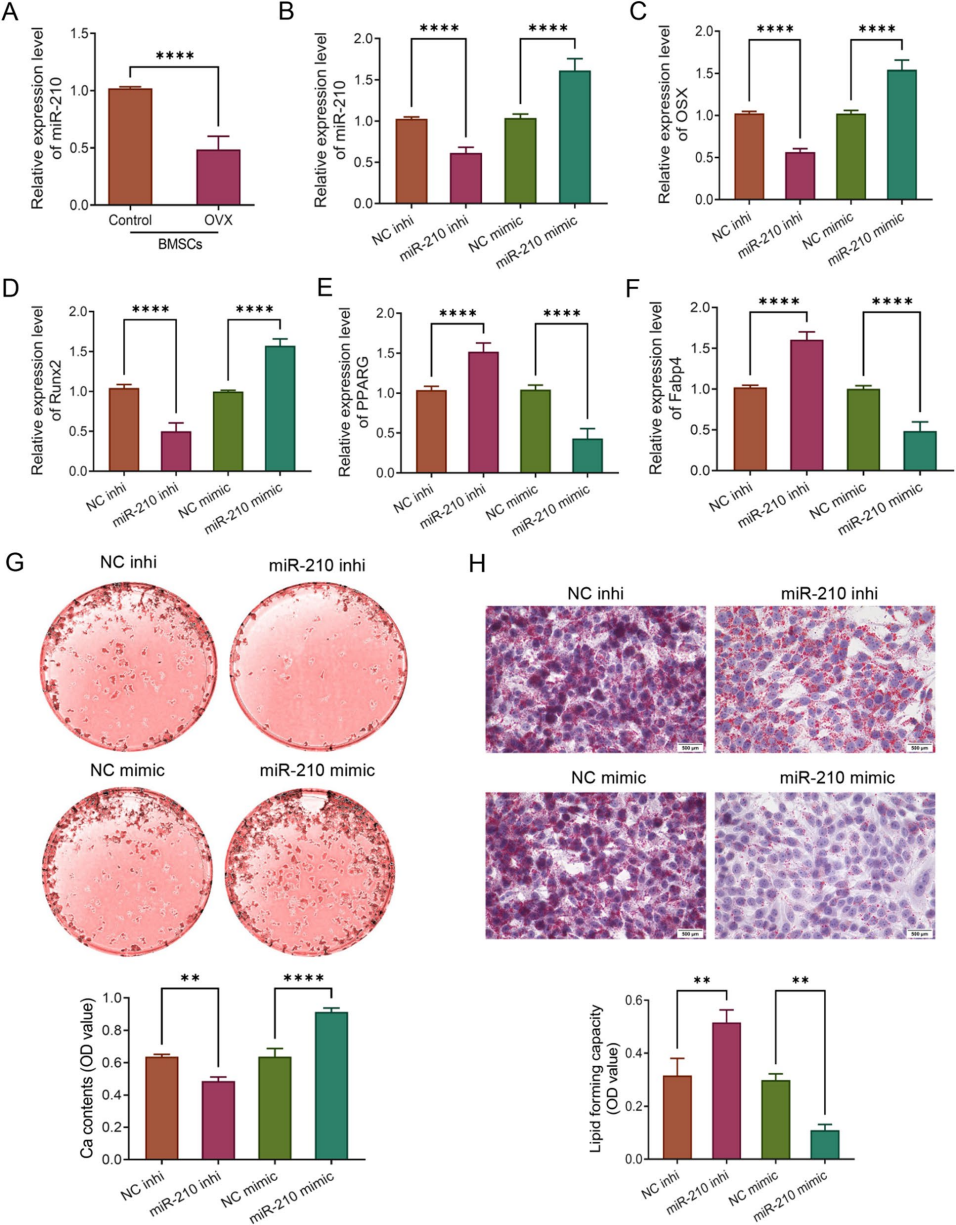
MiR-210 promotes osteogenic differentiation and inhibits adipogenic differentiation in BMSCs. **A** qRT-PCR to detect the expression levels of miR-210 in BMSCs of the Control and OVX groups, *n* = 5. **B**–**F**, qRT-PCR to detect the relative mRNA expression levels of miR-210 (**B**), OSX (**C**), Runx2 (**D**), PPARG (**E**) and Fabp4 (**F**) in BMSCs in the NC inhi and miR-210 inhi groups, NC mimic and miR-210 mimic groups, *n* = 5. **G** Alizarin red S staining to detect calcium mineralized nodule formation in BMSCs after knockdown or overexpression of miR-210 to further assess the osteogenic differentiation ability of the cells, *n* = 3. **H** Oil red O staining to detect the lipid formation in BMSCs after knockdown or overexpression of miR-210 to assess the adipogenic differentiation ability of the cells, *n* = 3. **P < 0.01, ****P < 0.0001. BMSCs: bone marrow mesenchymal stem cells; qRT-PCR: quantitative real-time polymerase chain reaction; OVX: ovariectomy; OSX: osterix; Runx2: Runt-related transcription factor 2; PPARG: peroxisome proliferator activated receptor gamma; Fabp4: fatty acid binding protein 4

Similar to the results in OVX rats, the expression level of miR-210 in the OVX rat-derived BMSCs in the OVX group was observably reduced compared to the Control group (Sham rat-derived BMSCs) (*P* < 0.0001, Fig. 2A, Additional file 1: Fig. S1). Subsequently, we overexpressed and knocked down miR-210 expression in BMSCs to further clarify the role of miR-210 in osteogenic differentiation and adipogenic differentiation of BMSCs, respectively. According to the results of qRT-PCR, the miR-210 inhi group showed significantly decreased expression levels of miR-210, Runx2 and OSX but increased expression of PPARG and Fabp4 compared with the NC inhi group. In contrast, the relative mRNA expression levels of miR-210 and osteogenic differentiation markers Runx2 and OSX in BMSCs were considerably elevated in miR-210 mimic group compared with those in the corresponding NC mimic group, while the expression levels of adipogenic differentiation markers PPARG and Fabp4 were notably reduced (*P* < 0.0001, Fig. 2B–F). In addition, the results of alizarin red S staining and oil red O staining also showed an obvious reduction in calcium mineralized nodule formation and a significant rise in lipid droplet formation in BMSCs after knockdown of miR-210, while BMSCs in the miR-210 overexpression group exhibited elevated Ca^2+^ deposition and reduced lipid formation (*P* < 0.01, Fig. 2G, H). All the above results indicated that overexpression of miR-210 significantly increased osteogenic differentiation and decreased adipogenic differentiation of BMSCs.

### MiR-210 targets and regulates EPHA2

**Fig. 3:**
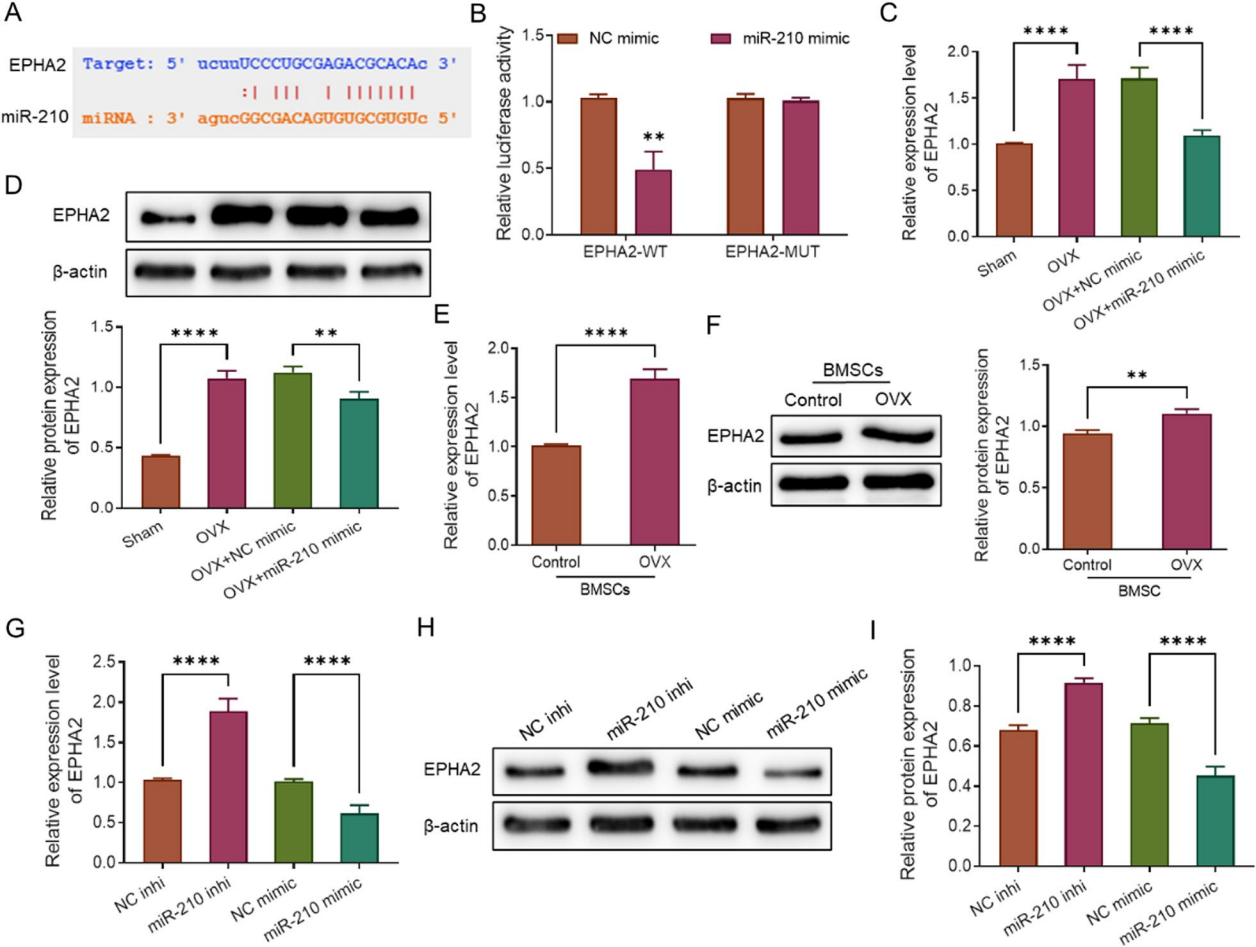
MiR-210 targets and regulates EPHA2. **A** The targeting sequence of miR-210 with EPHA2 predicted on starBase online website. **B** Dual luciferase reporter gene assay to verify the targeting relationship between miR-210 and EPHA2, *n* = 3. **C**, **D**, qRT-PCR and western blot to detect the relative mRNA (**C**, *n* = 3) and protein (**D**, *n* = 5) expression levels of EPHA2 in the Sham and OVX groups, the OVX + NC mimic and OVX + miR-210 mimic groups, respectively. **E**, **F**, qRT-PCR and western blot to detect the relative mRNA (**E**, *n* = 3) and protein (**F**, *n* = 5) expression levels of EPHA2 in BMSCs of the Control and OVX groups; **G**–**I**, qRT-PCR and western blot to detect the relative mRNA (**G**, *n* = 3) and protein (**H**, **I**, *n* = 5) expression levels of EPHA2 in BMSCs of NC inhi group and miR-210 inhi group, NC mimic group and miR-210 mimic group. **P < 0.01, ****P < 0.0001. EPHA2: ephrin type-A receptor 2; BMSCs: bone marrow mesenchymal stem cells; qRT-PCR: quantitative real-time polymerase chain reaction; OVX: ovariectomy

To further explore the molecular regulatory mechanism of miR-210, we first predicted the possible target genes of miR-210 through the online website starBase (https://starbase.sysu.edu.cn/). The prediction results displayed that miR-210 was able to target and bind to the 3'-UTR of EPHA2 (Fig. 3A). Moreover, the results of the dual luciferase reporter gene assay revealed that the cotransfection with miR-210 mimic effectively inhibited luciferase activity in cells of EPHA2-WT group (*P* < 0.01), while it had no effect on luciferase activity in the EPHA2-MUT group (Fig. 3B), confirming the targeting relationship between miR-210 and EPHA2. The expression levels of EPHA2 in rats and BMSCs of each group were subsequently examined. As a result, we found that the mRNA and protein expression levels of EPHA2 were much higher in the femoral tissues of rats in the OVX group compared with those in the corresponding Sham group (*P* < 0.0001), whereas the mRNA and protein expression levels were much lower in the femoral tissues of rats in the OVX + NC mimic group compared to the OVX + NC mimic group (*P* < 0.01, Fig. 3C, D). Similarly, a remarked increase in the mRNA and protein expression levels of EPHA2 in BMSCs was discovered in the OVX group compared with the Control group (*P* < 0.01, Fig. 3E, F). In addition, the mRNA and protein expression levels of EPHA2 were greatly increased or decreased in BMSCs with knockdown or overexpression of miR-210 compared with the corresponding transfected control groups (NC inhi group or NC mimic), respectively (*P* < 0.0001, Fig. 3G–I). Overall, miR-210 can target and regulate the expression of EPHA2.

### MiR-210 promotes osteogenic differentiation and inhibits adipogenic differentiation of bone marrow mesenchymal stem cells through down-regulation of EPHA2

**Fig. 4:**
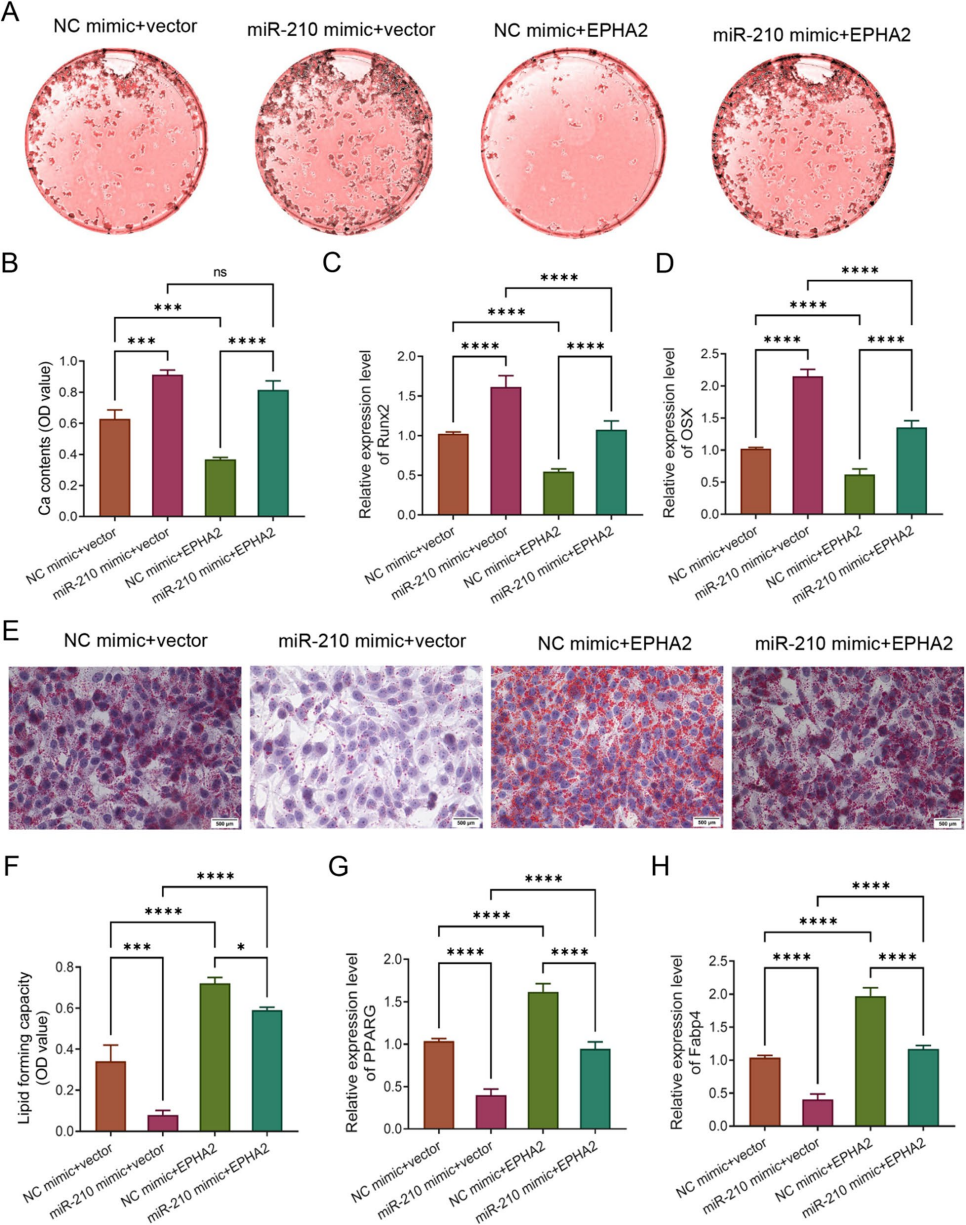
MiR-210 promotes osteogenic differentiation and inhibits adipogenic differentiation of BMSCs through downregulation of EPHA2. **A**, **B** Alizarin red S staining to assess the ability of calcium mineralized nodule formation in osteogenic differentiation of BMSCs in each group, *n* = 3. C/D, qRT-PCR to detect the relative mRNA expression levels of osteogenic differentiation markers Runx2 and OSX in BMSCs in each group, *n* = 5. **E**, **F**, oil red O staining to detect the ability of lipid formation in adipogenic differentiation in each group of BMSCs, *n* = 5; **G**, **H**, qRT-PCR to detect the expression levels of adipogenic differentiation markers PPARG and Fabp4 in each group of BMSCs, *n* = 3. *P < 0.05, ***P < 0.001, ****P < 0.0001. EPHA2: ephrin type-A receptor 2; BMSCs: bone marrow mesenchymal stem cells; qRT-PCR: quantitative real-time polymerase chain reaction; OSX: osterix; Runx2: Runt-related transcription factor 2; PPARG: peroxisome proliferator activated receptor gamma; Fabp4: fatty acid binding protein 4

Finally, we assessed whether miR-210 promoted osteogenic differentiation and inhibited adipogenic differentiation of BMSCs by downregulating EPHA2 through cotransfection of EPHA2 vector with miR-210 mimic into BMSCs. The results of alizarin red S staining and qRT-PCR disclosed a noble drop in the levels of calcium mineralized nodes and Runx2 and OSX in BMSCs of NC mimic + EPHA2 group compared with the NC mimic + vector group, indicating that EPHA2 played an inhibitory role in osteogenic differentiation of BMSCs. Relative to the miR-210 mimic + vector group, the ability of osteogenic differentiation was significantly inhibited in BMSCs of the miR-210 mimic + EPHA2 group, indicating that overexpression of EPHA2 could significantly weaken osteogenic differentiation of BMSCs promoted by miR-210 mimic (*P* < 0.0001, Fig. 4A–D). In contrast to osteogenic differentiation, the results of oil red O staining and qRT-PCR showed that upregulation of miR-210 greatly inhibited lipogenesis and reduced the levels of adipogenic differentiation markers PPARG and Fabp4, which implied that it inhibited adipogenic differentiation of BMSCs. However, upregulation of EPHA2 expression predominantly enhanced their adipogenic differentiation. Compared with the miR-210 mimic + vector group, the miR-210 mimic + EPHA2 group presented a marked rise in the adipogenic differentiation ability of BMSCs, suggesting that upregulation of EPHA2 could significantly improve the adipogenic differentiation ability inhibited by miR-210 (*P* < 0.0001, Fig. 4E–H). In short, miR-210 promoted osteogenic differentiation and inhibited adipogenic differentiation of BMSCs through down-regulation of EPHA2.

## Discussion

Postmenopausal OP is a persistent skeletal disease clinically characterized by reduced bone density and degeneration of bone trabecular architecture. The condition is typically age-related and is today one of the most prevalent diseases in China and the rest of the world [29]. Thus, it is crucial to investigate the mechanisms behind postmenopausal OP and to develop efficient preventative and therapeutic strategies [30]. Several studies have reported that miRNAs can be involved in regulating osteogenic differentiation and maintaining bone integrity in BMSCs by regulating gene expression [31, 32]. In this case, they may represent a promising potential therapeutic target for the treatment of postmenopausal OP. Because the decreased estrogen as a result of ovariectomy leads to increased bone resorption, bone turnover, and bone loss, OVX is commonly used to construct a model of postmenopausal OP [33]. In the present study, the levels of osteoblast marker genes ALP, Bglap and OSX were significantly downregulated in the femoral tissues of rats in the OVX group, suggesting that we successfully constructed postmenopausal OP rat models via OVX.

Additionally, we observed that miR-210 expression was obviously downregulated in both femoral tissues and BMSCs of OVX rats, and the levels of osteoblast markers ALP, OSX and Bglap significantly climbed in femoral tissues of OVX rats after miR-210 overexpression, indicating that miR-210 may promote bone formation in OVX rats. After overexpression of miR-210 in BMSCs, the osteogenic differentiation ability of BMSCs appeared to be significantly enhanced and the adipogenic differentiation ability was noticeably weakened, while the opposite was true after inhibition of miR-210. The role of miR-210 in increasing the osteogenic differentiation of BMSCs in OP rats was further supported by cellular research, which raises the possibility that miR-210 overexpression could be used as a therapeutic strategy to treat postmenopausal OP. Similar to the results of the present study, a sequencing study based on clinical samples from OP patients by Gu et al. showed that miR-210-3p expression was downregulated in the femur of OP patients. Liu et al. also found that miR-210 was involved in regulating bone formation in postmenopausal OP by promoting VEGF expression and osteoblast differentiation of BMSCs and ameliorating hormone deficiency [34]. In summary, miR-210 can act as a positive regulator of osteogenesis, and overexpression of miR-210 may improve OP via controlling osteogenic/adipogenic differentiation of BMSCs.

The well-known mechanism by which miRNAs function is to regulate the expression levels of downstream genes [35]. Through the online site starBase online site prediction and a series of basic experiments, we discovered that miR-210 could specifically target and suppress the expression of EPHA2. Further phenotype validation experiments demonstrated that the adipogenic capacity was significantly elevated, and mineralization capacity was markedly reduced in BMSCs after overexpression of EPHA2 alone, while overexpression of miR-210 along with EPHA2 decreased the adipogenic capacity and increased the mineralization capacity. These results suggest that miR-210 targets EPHA2 to enhance osteogenic differentiation and prevent adipogenic differentiation. EPHA2, a ligand in the EPHRIN-EPH signaling pathway, is a transmembrane glycoprotein with a molecular weight of about 130 kDa. It can be involved in tumor development through several classical signaling pathways such as protein kinase B (PKB), focal adhesion kinase (FAK), and mitogen-activated protein kinase (MAPK) [36]. EphA2 positive signaling to osteoblasts can inhibit bone formation and mineralization [37]. Interestingly, EPHA2 was found to be expressed in rat osteoblasts and its expression was elevated during osteogenic differentiation of rat BMSCs. Besides, EPHA2 can enhance the differentiation of multinucleated osteoblasts. All these results demonstrate that EPHA2 may play an important role in the process of osteogenic differentiation [38]. In addition, Liu et al. also found that 17β-estradiol could partially attenuate OVX-induced bone degeneration by inhibiting the ephA2 / ephrinA2 signaling pathway [39]. EphrinA2-ephA2 interaction promotes the initiation of bone remodeling by enhancing osteoclast differentiation and inhibiting osteoblast differentiation [40]. Thus, by affecting the ephrinA2-ephA2 signaling pathway, miR-210 may help to attenuate postmenopausal OP.

In summary, miR-210 promotes osteogenic differentiation and inhibits adipogenic differentiation of BMSCs by down-regulation of EPHA2 expression, thereby alleviating postmenopausal osteoporosis. However, only the role of miR-210 in osteogenesis/lipogenesis of BMSCs was explored in this study, and its role in osteoclast differentiation was not explored, so the study on bone resorption in bone homeostasis was missing. Further experiments are needed to clarify the role of miR-210 in bone resorption. In addition, this study, together with another article we published previously [22], explored the function and mechanism of miR-210 in postmenopausal OP. However, we point out the following differences between the two studies: (1) Mechanism differences. This study found that miR-210 plays a role in osteogenic/lipogenic differentiation of BMSCs by targeting EPHA2. A previous study found that the role of miR-210 in postmenopausal OP rats may be related to VEGF/Notch signaling pathway. (2) In the two studies, except for the simultaneous construction of the initial model rats, the other experiments and data were conducted and collected independently. (3) Different detection indicators. The former is mainly osteogenic/lipogenic differentiation in cell experiments, while the latter is mainly the level of osteogenic related markers in animal tissues. Overall, the above two studies provide us with a new perspective that miR-210 may form a complex regulatory network that directly or indirectly affects osteogenic differentiation of BMSCs through multiple factors. The mechanism by which miR-210 regulates osteogenic/osteoclast differentiation of BMSCs is unclear and further evidence is needed.

## Conclusion

Taken together, miR-210 expression is downregulated in femoral tissues and BMSCs of OVX rats, and its low expression is associated with reduced bone formation in OVX rats. More importantly, miR-210 promotes osteogenic differentiation and inhibits adipogenic differentiation of BMSCs by down-regulation of EPHA2 expression, thereby alleviating OP in menopausal rats. As a result, miR-210 serves as a new target for the diagnosis and treatment of OP in OVX menopausal rats since it is crucial for maintaining the osteogenic/adipogenic differentiation balance in BMSCs.

### Supplementary Information

**Additional file 1**. **Figure S1**: Identification of Bone marrow mesenchymal stem cells (BMSCs). Expression of the surface antigens CD11b, CD29 and CD90 on BMSCs was determined by flow cytometry.

## Data Availability

The datasets are available from the corresponding authors on reasonable request.

## References

[1] Sözen T, Özışık L, Başaran NÇ (2017). An overview and management of osteoporosis. Eur J Rheumatol.

[2] Fischer V, Haffner-Luntzer M (2022). Interaction between bone and immune cells: implications for postmenopausal osteoporosis. Semin Cell Dev Biol.

[3] Arceo-Mendoza RM, Camacho PM (2021). Postmenopausal osteoporosis: latest guidelines. Endocrinol Metab Clin.

[4] Migliorini, F., et al., *Fragility Fractures: Risk Factors and Management in the Elderly.* Medicina (Kaunas), 2021. **57**(10). 10.3390/medicina57101119 PMC8538459 34684156

[5] Migliorini F (2021). Effect of drugs on bone mineral density in postmenopausal osteoporosis: a Bayesian network meta-analysis. J Orthop Surg Res.

[6] Migliorini F (2021). Pharmacological management of postmenopausal osteoporosis: a level I evidence based - expert opinion. Expert Rev Clin Pharmacol.

[7] Cui Y (2022). A bone-targeted engineered exosome platform delivering siRNA to treat osteoporosis. Bioact Mater.

[8] Zhou Y (2020). Alterations in DNA methylation profiles in cancellous bone of postmenopausal women with osteoporosis. FEBS Open Bio.

[9] Sasaki A (2019). Mesenchymal stem cells for cartilage regeneration in dogs. World J Stem Cells.

[10] Galipeau J, Sensébé L (2018). Mesenchymal stromal cells: clinical challenges and therapeutic opportunities. Cell Stem Cell.

[11] Tian G-E (2019). Mechanoresponse of stem cells for vascular repair. World J Stem Cells.

[12] Agata H (2019). Intra-bone marrow administration of mesenchymal stem/stromal cells is a promising approach for treating osteoporosis. Stem Cells Int.

[13] Xu Z (2017). MiR-30a increases MDSC differentiation and immunosuppressive function by targeting SOCS 3 in mice with B-cell lymphoma. FEBS J.

[14] Yao Q, Chen Y, Zhou X (2019). The roles of microRNAs in epigenetic regulation. Curr Opin Chem Biol.

[15] Li Y (2019). miR-149-3p regulates the switch between adipogenic and osteogenic differentiation of BMSCs by targeting FTO. Mol Therapy-Nucleic Acids.

[16] Zheng HB (2021). MicroRNA-182 inhibits osteogenic differentiation of bone marrow mesenchymal stem cells by targeting Smad1. J Biol Regul Homeost Agents.

[17] Oliviero A (2019). MicroRNA in osteoarthritis: physiopathology, diagnosis and therapeutic challenge. Br Med Bull.

[18] Li ZH (2020). MiR-291a-3p regulates the BMSCs differentiation via targeting DKK1 in dexamethasone-induced osteoporosis. Kaohsiung J Med Sci.

[19] Zhuang Y (2022). Small extracellular vesicles derived from hypoxic mesenchymal stem cells promote vascularized bone regeneration through the miR-210-3p/EFNA3/PI3K pathway. Acta Biomater.

[20] Lončarić D (2019). Expression of miRNA-210 in human bone marrow-derived mesenchymal stromal cells under oxygen deprivation. Arch Biol Sci.

[21] Gu H (2019). Identification of differentially expressed microRNAs in the bone marrow of osteoporosis patients. Am J Transl Res.

[22] Ren LJ (2023). MiR-210 improves postmenopausal osteoporosis in ovariectomized rats through activating VEGF/Notch signaling pathway. BMC Musculoskelet Disord.

[23] Khedgikar V (2012). A standardized phytopreparation from an Indian medicinal plant (Dalbergia sissoo) has antiresorptive and bone-forming effects on a postmenopausal osteoporosis model of rat. Menopause.

[24] Zhou Q, Zhou L, Li J (2023). MiR-218-5p-dependent SOCS3 downregulation increases osteoblast differentiation inpostmenopausal osteoporosis. J Orthop Surg Res.

[25] Du Y (2022). Knockdown of CDC20 promotes adipogenesis of bone marrow-derived stem cells by modulating beta-catenin. Stem Cell Res Ther.

[26] Li W (2016). Regulation of the osteogenic and adipogenic differentiation of bone marrow-derived stromal cells by extracellular uridine triphosphate: The role of P2Y2 receptor and ERK1/2 signaling. Int J Mol Med.

[27] Li M (2022). Lipopolysaccharides affect compressed periodontal ligament cells via Eph–ephrin signaling. Oral Dis.

[28] Youngblood VM (2016). The ephrin-A1/EPHA2 signaling axis regulates glutamine metabolism in HER2-positive breast cancer. Can Res.

[29] Watts NB (2018). Postmenopausal osteoporosis: a clinical review. J Womens Health.

[30] Kim B, Cho YJ, Lim W (2021). Osteoporosis therapies and their mechanisms of action. Exp Ther Med.

[31] Li Y (2019). MicroRNA-92b-5p modulates melatonin-mediated osteogenic differentiation of BMSCs by targeting ICAM-1. J Cell Mol Med.

[32] Deng L (2018). Involvement of microRNA-23b in TNF-α-reduced BMSC osteogenic differentiation via targeting runx2. J Bone Miner Metab.

[33] Peng Z-Q (1997). Long-term effects of ovariectomy on the mechanical properties and chemical composition of rat bone. Bone.

[34] Liu X-D (2015). MicroRNA-210 is involved in the regulation of postmenopausal osteoporosis through promotion of VEGF expression and osteoblast differentiation. Biol Chem.

[35] O'Brien J (2018). Overview of microRNA biogenesis, mechanisms of actions, and circulation. Front Endocrinol.

[36] Kurose H (2019). Elevated expression of EPHA2 is associated with poor prognosis after radical prostatectomy in prostate Cancer. Anticancer Res.

[37] Matsuo K, Otaki N (2012). Bone cell interactions through Eph/ephrin: bone modeling, remodeling and associated diseases. Cell Adh Migr.

[38] Posthumadeboer J (2013). Surface proteomic analysis of osteosarcoma identifies EPHA2 as receptor for targeted drug delivery. Br J Cancer.

[39] Liu L (2018). 17beta-estradiol attenuates ovariectomy-induced bone deterioration through the suppression of the ephA2/ephrinA2 signaling pathway. Mol Med Rep.

[40] Shao J (2013). Eph–ephrin bidirectional signalling: a promising approach for osteoporosis treatment. J Med Hypoth Ideas.

