# Supplementary material for: MiR-210 promotes bone formation in ovariectomized rats by regulating osteogenic/adipogenic differentiation of bone marrow mesenchymal stem cells through downregulation of EPHA2
Source: J Orthop Surg Res. 2023 Oct 30;18:811. doi: 10.1186/s13018-023-04213-6 (PMC10617172; doi:10.1186/s13018-023-04213-6)

Supplementary figure 1. Identification of Bone marrow mesenchymal stem cells (BMSCs). Expression of the surface antigens CD11b, CD29 and CD90 on BMSCs was determined by flow cytometry.


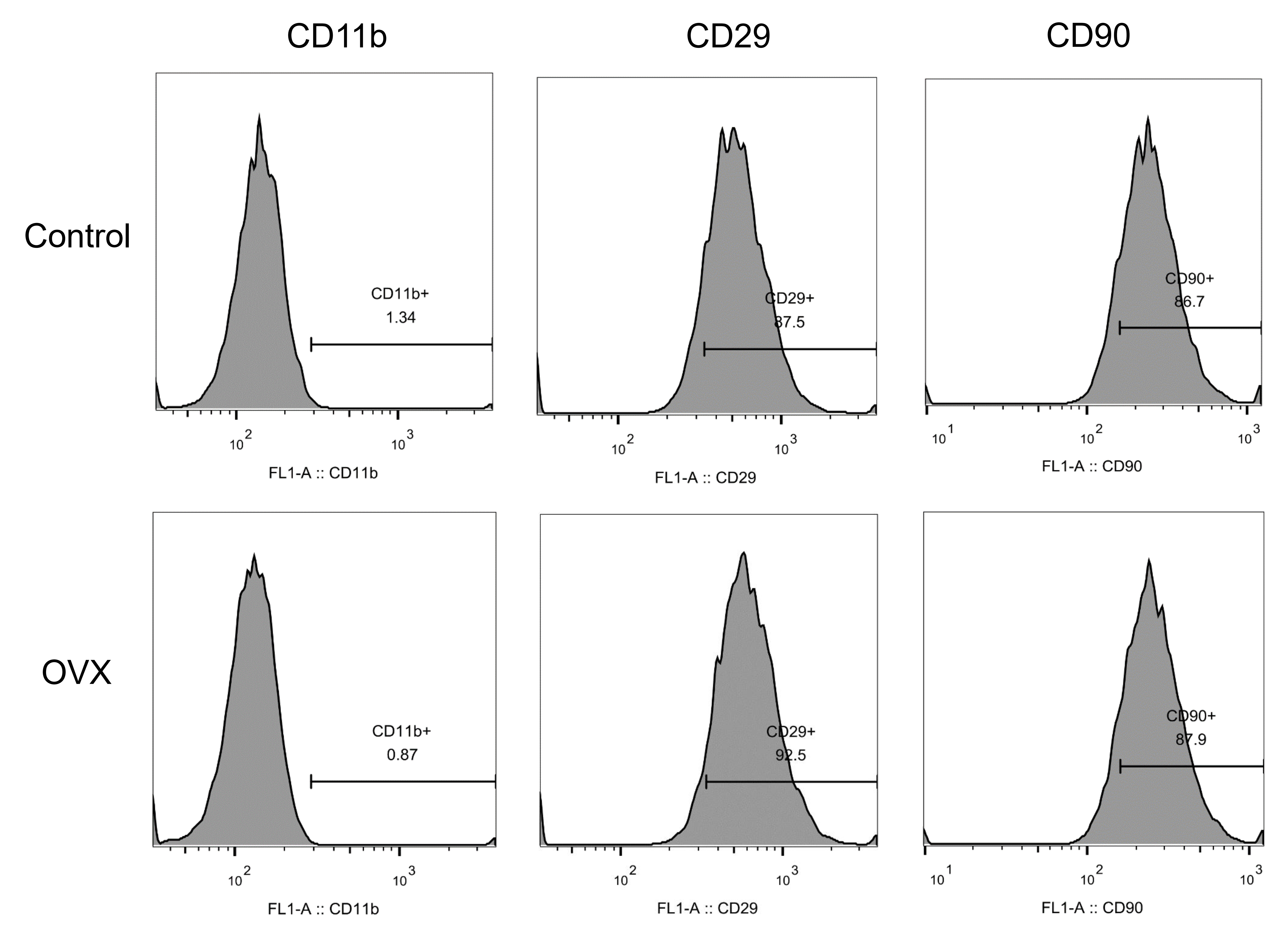

Supplement: Supplementary file 1 — Additional file 1. Figure S1: Identification of Bone marrow mesenchymal stem cells (BMSCs). Expression of the surface antigens CD11b, CD29 and CD90 on BMSCs was determined by flow cytometry. [file 13018_2023_4213_MOESM1_ESM.docx]
